# Case Report: Stretching the limits—late valvuloplasty for THV dysfunction following redo mitral valve-in-valve implantation

**DOI:** 10.3389/fcvm.2023.1288278

**Published:** 2023-10-31

**Authors:** David Meier, Georgios Tzimas, Mariama Akodad, Ali Husain, James Dundas, Julius Jelisejevas, Anson Cheung, Stephanie L. Sellers, Jonathon A. Leipsic, Philipp Blanke, David A. Wood, Janarthanan Sathananthan, John G. Webb

**Affiliations:** ^1^ Department of Cardiology, Lausanne University Hospital and University of Lausanne, Lausanne, Switzerland; ^2^ Cardiovascular Translational Laboratory, Providence Research and Centre for Heart Lung Innovation, Vancouver, BC, Canada; ^3^ Centre for Heart Valve Innovation, St. Paul’s Hospital, University of British Columbia, Vancouver, BC, Canada; ^4^ Centre for Cardiovascular Innovation, St Paul’s and Vancouver General Hospital, Vancouver, BC, Canada; ^5^ Institut Cardiovasculaire Paris Sud, Hôpital Privé Jacques-Cartier, Ramsay Santé, Massy, France

**Keywords:** mitral valve in valve, redo mitral valve in valve, late valvuloplasty, over-expansion, balloon dilatation

## Abstract

Late balloon valvuloplasty can be used to treat under-expansion-related transcatheter heart valve (THV) dysfunction. Whether this can be performed following redo-THV implantation is unknown. Herein, we report a case of a 72-year-old male presenting with symptomatic gradient elevation following redo mitral valve-in-valve implantation. The patient was successfully treated with late balloon valvuloplasty with gradient improvement. In conclusion, late valvuloplasty is effective even with several layers of valves. However, larger studies are required to clarify the role of this approach further.

## Introduction

Late balloon valvuloplasty with or without bioprosthetic valve fracture has been reported for addressing under-expansion-related transcatheter heart valve (THV) dysfunction, even several months after the index procedure (1). While these analyses have included valve-in-valve (VIV) cases, whether this can be effectively performed following redo-THV implantation remains unknown since the double stent layer might be less responsive to dilatation (2). Here, we illustrate a case where valvuloplasty was successfully performed several months following redo mitral VIV (MVIV).

## Case presentation

**Figure 1:**
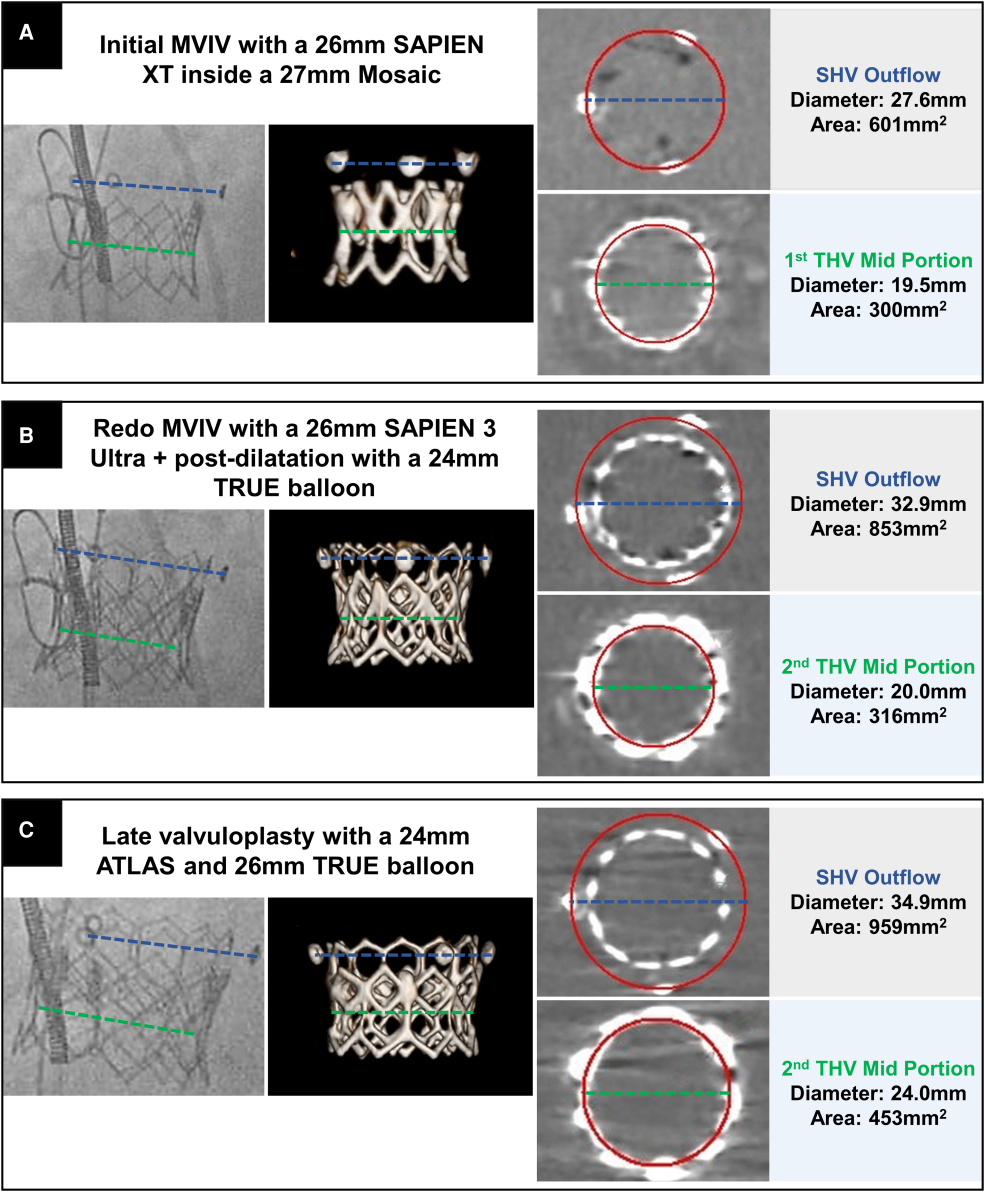
Fluoroscopic, CT volume-rendered, and multiplanar reformats CT images aligned with the THV illustrating the successive procedural steps with the initial MVIV (**A**), followed by redo-MVIV (**B**) and late valvuloplasty (**C**). The green line represents the mean diameter of the first (**A**) and second THV (**B,C**) in its mid-portion while the blue line illustrates the progressive flaring of the surgical heart valve (SHV) posts.

A 72-year-old male with severely reduced ejection fraction (19%) underwent redo-MVIV in 2022 for severely stenotic THV and NYHA III symptoms. He had initially undergone coronary artery bypass graft surgery with mitral valve replacement (27 mm Mosaic, Medtronic, USA) in 2004, followed by transapical MVIV (26 mm SAPIEN XT, Edwards Lifesciences, USA; Figure 1A) in 2013 for surgical valve degeneration. At that time, the THV was deployed at nominal volume and without surgical valve fracture.

For redo-MVIV, pre-procedural computed tomography (CT) showed no risk of neo-left ventricular outflow tract (LVOT) obstruction (predicted area of 14.5 cm^2^) due to the dilated left ventricle. In addition, the failed SAPIEN XT was found severely under-expanded (mid-portion at 19.5 mm, Figure 1A) without signs of valve thrombosis. Pre-dilatation using a 25 mm TRUE™ balloon (Bard Peripheral Vascular, USA) was thus performed, followed by transseptal implantation of a 26 mm SAPIEN 3 Ultra (S3) (Edwards Lifesciences, USA). The THV was then post-dilated using a 24 mm TRUE™ balloon without aiming for valve fracture or reaching very high pressure. Given the bi-directional flow and relatively large size of the atrial septal defect (ASD), the latter was closed using a 20 mm Amplatzer™ septal occluder device (Abbott Cardiovascular, Abbott Park, IL, USA). Despite the favorable discharge hemodynamic result [mean gradient (MG) 6 mmHg, heart rate (HR) 54 bpm], the S3 was found severely under-expanded on the pre-discharge CT scan with a mean diameter of 20 mm measured at the mid-portion of the THV (Figure 1B). The patient presented 1 month later with NYHA class III congestive heart failure symptoms and increased transvalvular MG (17 mmHg, HR 52 bpm). Since the patient was already anticoagulated with warfarin, this was felt unlikely to be due to valve thrombosis. Given the deterioration of the clinical status and the absence of any other treatment options, the patient was brought back (3 months after the initial procedure) for a balloon valvuloplasty to improve THV expansion. The procedure was performed under general anesthesia with transesophageal echocardiography guidance. Transseptal access was obtained above the ASD, and the valve was dilated using a 24 mm ATLAS balloon, followed by a 26 mm TRUE™ balloon at very high pressure (20 ATM for both) (Supplementary Video S1). The procedure was well tolerated despite the need for two long runs of rapid pacing (>20 s each), and the patient only needed transient inotropic support. At the end of the procedure, the second ASD was felt to be small with only left to right shunting and was treated conservatively. Fluoroscopy showed improved expansion confirmed on the CT scan with a mean diameter of 24 mm in the mid-portion of the THV. Interestingly, the CT scan also showed the overexpansion of the initial 27 mm Mosaic to almost 35 mm in diameter (Figure 1C). Given the multiple layers of valves and the frame design of the Mosaic, which makes visualization of the sewing ring difficult, whether fracture of the surgical valve had been achieved remained unclear.

**Figure 2:**
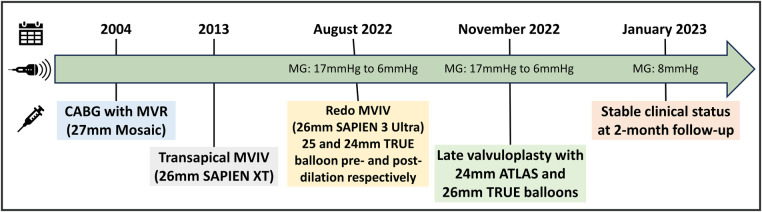
Timeline of case presentation with interventions performed and evolution of MG on transthoracic echocardiogram. CABG, coronary artery bypass graft surgery; MVR, mitral valve replacement.

The improvement in expansion was associated with a reduction in MG to 6 mmHg (HR 48 bpm) post procedure, and the patient was discharged the following day. During the 2-month follow-up, the patient reported improvement in clinical status (NYHA II). In addition, the transthoracic echocardiogram gradient remained stable at 8 mmHg (HR 55–60 bpm), with no signs of an increased LVOT gradient. The echocardiogram also showed moderate tricuspid regurgitation and elevated pulmonary artery systolic pressure of approximately 60 mmHg, which is stable compared with the pre-redo-MVIV. Figure 2 illustrates the procedural timeline and evolution of the transvalvular mitral MG.

## Discussion

This case report illustrates several important concepts relative to transcatheter mitral valve replacement.

First, redo-MVIV appears feasible in selected patients with acceptable hemodynamic results but might result in significant THV under-expansion if aggressive pre- and/or post-dilatation is not performed. However, it must be noted that the need for redo-MVIV remains relatively rare since MVIV is generally performed on elderly patients who rarely outlive their valves due to comorbidities (3).

Second, late balloon valvuloplasty can be effective despite multiple valve layers and can be used to treat under-expansion-related THV dysfunction. However, this requires long inflations at very high pressure that might be clinically poorly tolerated and with unknown long-term consequences on THV function. Moreover, it is possible that adequate pre-dilatation would facilitate the proper expansion of the redo-THV, similar to that observed in bench studies of redo transcatheter aortic valve replacement (TAVR) (2).

Finally, post-dilatation of several layers of valves may lead to marked flaring of the surgical bioprosthesis posts, which may increase the risk of LVOT obstruction. Indeed, CT simulation for pre-procedural neo-LVOT area prediction depends on assuming a cylindrical geometry projected over the existing bioprosthesis into the LV. If the valve outflow becomes flared post-dilatation, then the resultant valve instead has a conical geometry, with the wider end reducing the true neo-LVOT area compared with that predicted on the CT scan. Similarly, in the aortic VIV TAVR arena, post-dilatation or valve fracture causing under-estimation of coronary obstruction risk has been recently reported (4). Our case highlights that further valve modification post-deployment may also affect procedural risk in the mitral space beyond that predicted by standard pre-procedural CT assessment. Structural imaging and interventional clinicians should be aware of this when balloon valvuloplasty or valve fracture is being considered.

## Conclusion

Late balloon valvuloplasty is feasible in selected patients following redo-MVIV. However, further studies are required to assess the effectiveness and implications of high-pressure valvuloplasty following redo-THV implantation.

## Data Availability

The original contributions presented in the study are included in the article/**Supplementary Material**, further inquiries can be directed to the corresponding author.
